# Environmental Applications of Electromembrane Extraction: A Review

**DOI:** 10.3390/membranes13080705

**Published:** 2023-07-28

**Authors:** Linping Shi, Mantang Chen, Ge Zhao, Xiaoyu Wang, Meijuan Fan, Ruihong Liu, Fuwei Xie

**Affiliations:** 1 College of Chemistry, Zhengzhou University, Science Avenue #100, Zhengzhou 450001, China; slphxy@163.com; 2 Zhengzhou Tobacco Research Institute of CNTC, Fengyang Street #2, Zhengzhou 450001, China; zhaogztri@163.com (G.Z.); wangxiaoyu@iccas.ac.cn (X.W.); fanmj1018@aliyun.com (M.F.); liuruihong1006@163.com (R.L.)

**Keywords:** electromembrane extraction, environmental analysis, environmental remediation, inorganic ions, organic pollutants

## Abstract

Electromembrane extraction (EME) is a miniaturized extraction technique that has been widely used in recent years for the analysis and removal of pollutants in the environment. It is based on electrokinetic migration across a supported liquid membrane (SLM) under the influence of an external electrical field between two aqueous compartments. Based on the features of the SLM and the electrical field, EME offers quick extraction, effective sample clean-up, and good selectivity, and limits the amount of organic solvent used per sample to a few microliters. In this paper, the basic devices (membrane materials and types of organic solvents) and influencing factors of EME are first introduced, and the applications of EME in the analysis and removal of environmental inorganic ions and organic pollutants are systematically reviewed. An outlook on the future development of EME for environmental applications is also given.

## 1. Introduction

Serious environmental pollution has attracted increasing attention because of its toxicity and bioaccumulation. It is highly challenging to accurately identify environmental pollutants, especially at trace levels, without pre-treatment procedures. The most popular technique for completely extracting organic chemicals from solid samples is called soxhlet extraction, and it was created in 1879. However, there are several drawbacks to Soxhlet extraction, including the length of time needed (usually 16 to 24 h) and the amount of solvent required (often 100 to 500 mL). Pressurized liquid extraction (PLE) was introduced in 1996 as an alternate extraction method and had the advantage of decreased extraction time. However, when using a nonpolar extraction solvent, wet samples must be dried before analysis, which is a disadvantage of PLE for all classes of analytes [1]. Researchers have been compelled to develop novel, effective, economical, environmentally friendly, and secure extraction procedures as a result of these limitations.

Liquid-phase microextraction techniques (LPME) use a liquid medium (ionic liquids, single organic solvents, or mixed organic solvents) as the extraction phase. To begin, 100 μL or less of solvent is loaded in a support, such as hollow fiber (HF) or membrane, by specific techniques (coating or impregnation). Liquid-phase microextraction techniques allow the separation and enrichment of trace targets from complex matrices. Zhao and Lee [2] presented the HF-LPME in 2002 to address the shortcomings of LPME, including unstable droplets, easy to fall off from the tip of the needle, and poor repeatability. In HF-LPME, target analytes are extracted from aqueous samples and transferred to a supported liquid membrane (SLM) maintained in the pores in the wall of a small porous hollow fiber, then to an acceptor phase inside the hollow fiber’s lumen. The main benefits of these techniques are their low cost, ease of use, small sample volume, rapidity, extremely low solvent consumption, high enrichment factor, and reduced waste generation. The electromembrane extraction (EME) proposed by Pedersen-Bjergaard and Rasmussen in 2006 further improved the extraction efficiency and extraction time on this basis [3].

The classical EME device applies an electric field at two electrodes in the sample solution and the hollow fiber lumen based on the HF-LPME device [4]. Another type of EME device is the two-phase EME. A plastic centrifuge tube (2 mL) was used as the donor chamber and a 1000 μL pipette tip as the acceptor chamber, with a polypropylene membrane sealed at the bottom of the tip. Two platinum electrodes were embedded in the donor and acceptor with an average distance of 1 cm between the electrodes [5]. In EME, charged analytes are extracted from the aqueous sample solution across an SLM and into an aqueous acceptor phase while being subjected to an electrical field. The acceptor phase was then collected for chemical analysis. The SLM is a porous polymeric support membrane that has an organic solvent immobilized by capillary forces. The use of nanostructured materials, biopolymer membranes, and gels as supporting liquid membranes has also been reported. SLM is selective for the extraction of target analytes and therefore provides excellent sample clean-up capability [6]. An electrical potential (DC) sustained over the SLM, with one electrode positioned in the sample and the other electrode positioned in the acceptor phase, serves as the driving force for the extraction [3]. The pH of the sample and the acceptor solution must both be neutral or acidic to preserve the protonated state of the cationic analytes (basic chemicals) during extraction. The target analytes are therefore vulnerable to electrokinetic migration because they are positively charged species. The anode is in the sample, while the cathode is in the acceptor solution. For extraction of anionic analytes (acid substances), the pH in the sample and the acceptor solution is neutral or alkaline to maintain the analytes negatively charged. Thus, the electric field is in the opposite direction.

Most articles focus on the analysis of inorganic ions and organic pollutants in the environment. A few articles explore the application of EME in environmental remediation. In this review, we present an overview of the application of EME in the environment in recent years. Finally, critical insights into the future trends of EME are provided.

## 2. Basic Experimental Equipment and Parameters

The standard instrumental setup is a modification of the HF-EME device. The sample vial is pipetted with donor solution and set on an agitator. In order to create the SLM, the HF extraction unit is briefly impregnated with the proper organic solvent, and the HF lumen is then filled with the acceptor solution. The HF is added to the donor solution, the electrodes are inserted with one electrode in the donor solution and the other in the acceptor solution, and the electric potential is turned on. To achieve effective analyte replenishment at the donor/SLM phase contact, which is crucial for high extraction recovery and fast extraction speed, agitation of operational solutions is preferable. In the actual example, basic analytes quickly reach the SLM/acceptor phase contact by migrating into and across the SLM as charged species. Utilizing an acidic acceptor phase (such as diluted acid or an acidic buffer) allows the analytes to be released into the acceptor solution while maintaining their protonated state. Analytes can be gradually preconcentrated in the acceptor solution thanks to the protonation of basic analytes, which prevents their back-extraction into SLM. Due to their respective lack of positive charges, neutral and acidic matrix chemicals do not participate in the EME process, and even if they were to diffuse into the SLM, they would not significantly liberate any acidic acceptor solution. After the extraction is finished, the acceptor solution is collected and put into a vial for analysis using high-performance liquid chromatography (HPLC), gas chromatography (GC), capillary electrophoresis (CE), etc.

Success in EME depends heavily on the mass transfer of the SLM [7]. The standards for quality SLM candidates include (a) high polarity-polarizability, (b) low vapor pressure, (c) high affinities and robust chemical interactions between the analyte and the organic solvent, and (d) low water solubility [8]. Researchers have experimented with many methods to increase the mass transfer through the SLM in EME. Hasheminasab and Fakhari [9] decorated hollow fibers (HF) with carbon nanotubes (CNTs) and thus increased the overall analyte partition coefficient over the SLM. Huang et al. [10] discovered that the SLM volume was crucial for stable EME and that raising the SLM volume (thickness) might lower the extraction current during EME.

The most frequently used and effective solvent in EME for nonpolar (log P > 2) basic drugs has been 2-nitrophenyl octyl ether (NPOE). For protonated basic compounds (log P > 2), hydrogen bond interactions have been proposed as the primary mechanism of solvation. Recently, several NPOE substitutes have been proposed. HosseinyDavarani et al. [11] selected 2-ethyl hexanol as a solvent for SLM, and the study showed that the recovery was up to 85%. Drouin et al. [12] investigated 22 organic solvents as novel loading liquid membranes and showed that 2-nitrophenyl pentyl ether (NPPE) extracted polar metabolites with the highest efficiency, high extraction yield (EY) and reproducibility, and with large coverage of the extracted metabolites. The optimized experimental parameters were successfully used for the extraction of precipitated plasma samples. Pure NPOE is ineffective for more polar basic chemicals (log P < 2) as mass transfer from the sample and into the SLM is inefficient. An ion pair reagent, such as di-(2-ethylhexyl) phosphate (DEHP) or tris-(2-ethylhexyl) phosphate (TEHP), was added to the organic solvent to produce ion pairs with the analytes to improve the transport of polar drugs through SLM. However, the current in the system is increased by the addition of DEHP. Tributyl phosphate (TBP) and di(2-ethylhexyl) phosphite hydrogen salts are used as substitutes for the polar basic analytes in EME. Effective SLM solvents have high hydrogen-bond basicity (very high Kamlet–Taft β-value), high polaritypolarizability (high Kamlet–Taft π*-values), no hydrogen-bond acidity (zero Kamlet–Taft α-values) and contain alkyl groups equivalent to 12–18 carbon atoms [13]. For the extraction of acidic medicines, NPOE is ineffective as the SLM. Strong hydrogen bond acidity in the SLM solvent is necessary for the EME of acidic materials, and typical SLMs are made of aliphatic alcohols such as 1-octanol and 1-heptanol. Aliphatic alcohols are insoluble in water and tend to impregnate films.

Polypropylene (PP) is often chosen as the material used in EME procedures [14]. However, this material is plastic and disposable, and is harmful to the environment. Therefore, these drawbacks have been overcome by modifying the liquid membrane or commercial support. Some additives such as carrier agents, nanoparticles (NPs), and molecularly imprinted polymers (MIPs) are usually added to change the chemical composition of SLMs [15,16]. In addition, the use of agarose gels instead of conventional polymeric porous membranes in EME and without the use of organic solvents has also been reported [17]. Agarose is considered to be a green material. Another recent development in EME environmental applications is the application of green biodegradable membranes instead of classic SLMs. Materials derived from biopolymers (such as agarose or chitosan) are biodegradable and sustainable materials and have recently gained a lot of attention in EME [14,17,18]. These materials, which are obtained from natural resources, are environmentally friendly, and have low economic costs.

Huang et al. [19] proposed a time-dependent transient model to simulate the mass transfer of SLM in EME. This model is mostly based on three hypotheses: (a) the SLM residence time of the analyte produces a lag time, (b) the transport via the SLM is the extraction stage that determines the rate, and (c) there is only one direction of transport through the SLM [20]. This model confirms that the voltage applied across the liquid membrane is the main operating parameter in EME. The voltage is typically provided between 5 and 600 V. Recovery values decline at higher voltages due to electrode bubble formation and system instability [3]. Time is another important parameter that affects recovery. There are several possibilities for the decrease in recovery over time: (a) unstable current in the system, (b) elevated pH in the acceptor phase, (c) diffusion of analytes into the donor phase, and (d) loss of a small amount of artificial liquid film [20]. Typically, the extraction process takes no longer than 15 min.

During EME, pH can shift in both the sample and the acceptor phases. It has been shown that a decrease in the ratio of total ion concentration on the donor side to total ion concentration on the recipient side increases ion flux [21]. The analytes are extracted across the SLM in EME, with a positive electrode placed in the sample and a negative electrode placed in the acceptor solution to quickly extract basic analytes. The pH in the sample and acceptor solutions must be low to ionize the analytes. The pH of the donor and acceptor solutions was alkaline during the extraction of acidic drugs. Acceptor solutions with high concentrations of weak organic and strong mineral acids can produce stable EME performance [22].

Stirring speed increases mass transfer and decreases the thickness of the bilayer around the SLM, improving extraction kinetics and extraction efficiency [23]. The donor solution is typically vigorously stirred or agitated to ensure an efficient, long-lasting interaction between the analyte ions and the SLM surface. Typically, stirring is maintained at a speed of 0–1250 rpm [23]. Due to the production of bubbles in the donor and acceptor phases, as well as the leaking of the organic solvent from the SLM, extraction recovery is reduced at higher stirring rates.

Basically, the diffusion coefficient also increases with increasing temperature, but the driving force of the system decreases with increasing temperature [21]. To assess the impact of sample solution temperature, Rahmani et al. [24] used a range of sample solution temperatures (25–60 °C) during the extraction process while monitoring the extraction recovery and membrane current intensity. The results suggest that when the temperature of the donor solution rises, extraction recovery follows a declining pattern. Temperatures up to 40 °C can hasten the extraction process, while those above 40 °C can partially degrade the SLM [25].

## 3. EME for Environmental Analysis

Environmental analysis refers to the process of measuring the possible pollutants and sources of pollution in an environment using specific technical means, which can provide accurate and critical reference information and a basis for environmental protection work. EME is a common pretreatment method for environmental samples with complex matrices that cannot be directly detected by analytical instruments. Most references in this review use water samples as an example to explore the feasibility of EME in the environmental analysis.

### 3.1. Determination of Inorganic Ions by EME

Inorganic ionic pollutants, including dichromate, perchlorate, and heavy metal cations, are pervasive in the environment and pose substantial risks to people and the natural world. For example, when inhaled or placed in contact with skin, Cr(VI) damages the kidney and liver and is more difficult to treat than other pollutants that decompose through enrichment [26]. Arsenate is a highly biotoxic substance that can lead to poor nutritional growth in plants, and the introduction of arsenate into the human body alters the normal metabolism of cells [27].

Tan et al. [28] designed and fabricated a concentric electromembrane extraction preconcentration device for the determination of ${\mathrm{NO}}_{3}^{-}$ and ${\mathrm{SO}}_{4}^{2-}$ content from a soil slurry without additional treatment using fused deposition modeling 3D printing technique. A smaller, hemispherical 3D-printed porous membrane acceptor vial was placed within the larger, hemispherical electrode sample vial made of conductive polylactic acid filament. The system is efficient at ion extraction from numerous samples into a smaller acceptor solution. Fluorescein was used to image the extraction process in order to demonstrate electromembrane extraction, with preconcentration rates of 8.33 × 10^−7^ mol/L per second at 120 V and close to 95% recovery. The enrichment factor was between 36–44 and the LOD was between 1.6 × 10^−7^–1.8 × 10^−7^ mol/L. The ${\mathrm{NO}}_{3}^{-}$ and ${\mathrm{SO}}_{4}^{2-}$ concentrations extracted and quantified from the soil slurry were within the range of values typically reported in soil samples. For strong acid anions, the device showed good recovery, but it performed poorly for weak acid anions.

Khan et al. [29] reported the effective EME of Cu(II) in different environmental water samples followed by Red-Green-Blue (RGB) detection. The applied voltage, the extraction time, the composition of the SLM, the pH of the acceptor/donor phases, and the stirring rate are the effective parameters that have been optimized for the extraction efficiency of EME. 1-octanol was adopted as the SLM and 0.1 mol/L HCl was used as the donor and acceptor solutions. A voltage of 50 V was used as the driving force, the system was agitated at 400 rpm, and the extraction time was 15 min. The relative recoveries were in the range of 93–106%. The enrichment factor was 100. The method provides a rapid, simple, and effective determination of Cu(II).

Nojavan et al. [30] discussed the use of free liquid membranes (FLM) for the micro-electromembrane extraction (µ-EME) of Cr(VI) from wastewater samples. Electrothermal atomic absorption spectrometry was used to calculate the amount of Cr(VI). It was optimized for FLM and acceptor solution types, donor and acceptor solution pH, electrical potential, and FLM thickness. The SLM was composed of 1-octanol containing 5% methyl trialkyl-ammonium chloride with a thickness of 1 mm, and the extraction time was 5 min. In addition, 0.5 mol/L NaClO_4_ and 0.1 mol/L HCl were used as the acceptor and donor phases, respectively. The driving force for the extraction was 75 V. According to the conventional addition approach, relative recoveries of the spiked Cr(VI) in the samples ranged from 73.8 to 85.1%. The calculated limit of detection was less than 0.06 ng/mL. μ-EME is a promising option for the selective extraction of Cr(VI).

Kaya [31] made improvements to the membrane process to improve the long-term use and stability of the membrane. Amine functional reduced graphene oxide (rGO-fNH_2_) and modified membrane including 51.85% NPOE, 29.63% cellulose triacetate, and 18.52% rGO-fNH_2_ were synthesized. In an experiment with the constant current instead of constant voltage, the membrane stability was still above 90% after 30 repetitions. Improvements made to the membrane process have improved the long-term use and stability of the membrane. However, the use of electricity during the experiments can damage the membrane structure.

Tabani et al. [32] chose positively charged Cr(III) and negatively charged Cr(VI) as model analytes, and investigated the effect of electroendotropic (EEO) flow phenomenon on gel electroemulsion extraction (G-EME). A low-cost, simple-to-use reading platform known as a microfluidic paper-based analytical device was used to quantify both compounds. By using G-EME for the determination of chromium in water samples, the instrument has a high level of environmental friendliness, improved sensitivity, and higher analytical performance by enrichment extraction. The recoveries were 87% for Cr(III) and 75% for Cr(VI). The LODs were 0.5 and 0.7 ng/mL for trivalent and hexavalent chromium, respectively.

Fashi et al. [33] reported the EME combined with microvolume UV-Vis spectrophotometric detection of mercury in water and fish samples. For the electromembrane extraction of mercury, 1-octanol and 2% (*v*/*v*) DEHP was chosen as the appropriate membrane composition. The pH of the donor solution (0.01 mol/L NaOH) was 7.0 and the pH of the acceptor solution (0.01 mol/L NaOH) was 3.0. The driving force for the extraction was 12 V, and the unit was agitated at 700 rpm for 10 min. The LOD was found to be 0.7 µg/L and 12 µg/kg in water and fish samples, respectively. The repeatability and reproducibility of the procedure were excellent.

Silva et al. [34] reported the simultaneous EME of six trace metal cations (Cu^2+^, Zn^2+^,Co^2+^, Ni^2+^, Pb^2+^, Cd^2+^) from saline samples. Metals in acceptor solutions were identified using CE with capacitively linked contactless conductivity detection (C^4^D). The SLM was composed of 1-nonanol containing 1% (*v*/*v*) di-2-ethylhexyl phosphoric acid (DEHPA). The driving force was 10 V, and the extraction time was 20 min. The extraction unit was stirred with a stirring rate of 750 rpm. The pH of the donor solution was 5.0 and the acceptor solution was 1 mol/L acetic acid. The approach demonstrated high linearity in the concentration range of 5 × 10^−7^–10^−6^ mol/L and reproducibility between 8 and 21.8% (*n* = 5). The LOD of this method is better than 2.6 × 10^−9^ mol/L.

Table 1 summarizes the applications of EME in the determination of inorganic ions.

### 3.2. Determination of Organic Pollutants by EME

Organic pollutants including cationic quaternary ammonium salts, anionic dyes, phenolics, drugs, and additives are highly toxic, mutagenic, carcinogenic and difficult to degrade. The direct discharge of untreated pollutants into water bodies affects the growth of plants and animals and human health.

Mollahosseini et al. [5] reported a novel flat plate membrane for extracting DEHP from the aqueous phase using a two-phase EME technique. The electrospinning technique was adopted to create polyacrylonitrile (PAN)/polydimethylsiloxane (PDMS) and PAN/PDMS/titanium dioxide (TiO_2_) membranes. When the electrospun nanofibers contained 3.3% (*w*/*v*) TiO_2_ nanoparticles, the extraction efficiency was best. After increasing the TiO_2_ nanoparticle concentration to 4.4% (*w*/*v*), the TiO_2_ nanoparticles aggregated inside the nanofibers and formed beads, which would reduce the specific surface area of the electrospun nanofibers and lead to a decrease in the performance of the membrane. The extraction time was 20 min, and the donor pH was 5.0. A small volume of 1-octanol (0.8 mL) was used as the acceptor solution. DEHP was detected by UV-Vis spectrophotometry. In the concentration range of 20–1000 ng/mL, the relative standard deviation (RSD), the limit of detection (LOD), and the limit of quantitation (LOQ) were 8%, 5 ng/mL, and 16.67 ng/mL, respectively. Application on an industrial scale of the suggested technique is possible.

Guo et al. [64] used double surfactants-assisted electromembrane extraction by Tween^®^20 and alkylated phosphate for the determination of cyromazine and melamine in surface water, soil, and cucumber. To begin, 6 × 10^−4^ mol/L Tween^®^20 and alkylated phosphate were added to the donor phase and the SLM, respectively. Capillary electrophoresis with capacitively coupled contactless conductivity detection was used to directly assess the extract. The SLM was composed of 80% NPOE, 10% DEHP, and 10% TEHP. The pH of donor solution and acceptor solution were 5.5 and 3.0, respectively. The extraction process took 20 min with a stirring speed of 1000 rpm and an extraction voltage of 60 V. The average recoveries ranged from 93.3 to 112%.

Rezaei et al. [65] reported a dual gel EME of model basic and acidic dyes from a wastewater sample. The extracts did not require organic solvents and the membrane components are highly degradable. The device was completed with green analytical chemistry technology standards. The positively charged brilliant green (BG) and negatively charged reactive red (RR) moved selectively from a 7.0 mL aqueous sample (pH 7.0) to the 500 µL cathodic (pH 4.0) and 500 µL anodic (pH 9.0) aqueous acceptor phases. The driving force was 50 V, and the extraction time was 20 min. Under ideal circumstances, the LODs were 0.020 and 0.025 ng/mL, and the extraction recoveries were 75–78% and 67–71% for BG and RR, respectively. For both model analytes, linearity was found to be between 0.06 and 1.0 g/mL with a correlation coefficient above 0.996.

Balchen et al. [66] reported the EME of 37 different peptides from a 500 µL aqueous sample. The extractability of peptides was linked to the highly variable physical-chemical properties of peptides by three different SLMs. SLMs with different organic solvents (pure eugenol, 1-octanol/di-isobutylketone/DEHP, 1-octanol/15-crown-5 ether) were tested. This research has shown that a variety of peptides can be successfully extracted with EME under various conditions.

HosseinyDavarani et al. [11] used a virtual rotating SLM for EME of model analytes from wastewater and human urine samples. Unique electrode assemblies that included a core electrode submerged inside the lumen of the SLM and five counter electrodes encircling the SLM were used to achieve the virtual (nonmechanical) rotating of the SLM. The electrical potential was distributed among five counter electrodes in a revolving pattern using a specific electronic circuit. 2-ethyl hexanol was used as the SLM. The driving force for the extraction was 200 V and the extraction time was 15 min. The RSDs ranged from 12.6 to 14.8%, and recoveries ranged from 38 to 85%.

Asadi et al. [67] prepared polyacrylamide gels as a novel membrane in EME for the extraction of three model essential drugs (pseudoephedrine, lidocaine, and propranolol) by HPLC-UV. A polyacrylamide gel membrane with a 2 mm thickness and a pH of 1.5 was created. It contains 3.0% (*w*/*w*) bisacrylamide and 12.0% (*w*/*v*) acrylamide. The pH of the donor phase was 7.0 and the acceptor phase was 4.0. The driving force for the extraction was 85 V and the extraction time was 28 min. The ranges of the limits of quantification and detection were 1.0–20.0 ng/mL and 0.3–6.0 ng/mL.

Table 2 summarizes the applications of EME in the determination of organic pollutants.

## 4. EME for Environmental Remediation

Various methods, such as adsorption, photocatalysis, coagulation-precipitation, reverse osmosis, air flotation, ultrasonication, chemical oxidation, incineration, biochemistry and EME, have been developed for the treatment of wastewater pollutants. As a new technique, EME provides an innovative strategy for environmental remediation.

### 4.1. Removal of Inorganic Ions by EME

Golubenko and Yaroslavtsev [93] obtained a series of modified anion-exchange membranes to form a pair of thin charged layers by utilizing the surface-sulfonated graft anion-exchange membranes. The formation of the interfacial layer significantly improved the selectivity of Cl^−^/${\mathrm{SO}}_{4}^{2-}$ and the resistance to humic acid adsorption. In electrodialysis, the best surface-sulfonated anion-exchange membrane has a high Cl^−^/${\mathrm{SO}}_{4}^{2-}$ selectivity of up to 6.4. It was established that the current density affects the Cl^−^/${\mathrm{SO}}_{4}^{2-}$ selectivity of the surface-sulfonated anion-exchange membrane during the desalination process. The obtained membranes exhibit antifouling characteristics towards high-molecular-weight pollutants such as humic acids.

La Cerva et al. [94] proposed two coupled models for the Reverse Electrodialysis (RED)-Reverse Osmosis (RO) and Electrodialysis (ED)-RO systems of seawater desalination. In the electro-membrane pre-desalting process, the RED-RO consumed less energy than the standalone seawater reverse osmosis (SWRO) for a wide range of external voltages. The cost of ED-RO desalination was higher than that of SWRO. The RED-RO case offered significant cost savings and had strong industrial application potential.

Gurreri et al. [95] combined an electric membrane process with RO technology to desalinate real seawater in a pilot plant. The integrated plant recovered approximately 40% of its total water. In the ED-ED-RO, ED-RO, and shortcut reverse electrodialysis (scRED)-assisted reverse electrodialysis (ARED)-RO setups, ED, scRED, and ARED were investigated. When efficient ERDs are considered, the energy usage of SWRO was 20% lower than the integrated plant’s minimal value. Coupling of electrolytic membrane processes with RO processes may develop alternative, competitive industrial membranes for seawater desalination.

Kuban [96] proposed a novel method for rapid and efficient desalination of tiny volumes of brine samples using multiphase microelectroelectric membrane extraction (μ-EME). The aqueous sample was the center solution sandwiched between two organic free liquid membranes (FLMs) and two aqueous extraction solutions contained in a disposable, clearly transparent extraction device containing microliter quantities of five immiscible solutions. Target analytes were selectively prevented from migrating across the organic phase by two FLMs, and analytes were kept in the sample. 1-pentanol was used as the SLM, and extraction solutions were composed of 15 µL deionized water and 0.1 mol/L NH_4_OH. The driving force was 300 V and the extraction time was 60 min. From samples with physiological NaCl values, more than 99.3% NaCl was removed. Each phase in the extraction unit can have a unique volume, length, and composition. Simply changing the volume of the FLM (decrease or increase) allows for the desalination of samples with different salinities, resulting in faster and more stable desalination of low and high-salinity samples, respectively. Sample desalination and targeted retention of specific analytes can be achieved by fine-tuning the composition of the FLMs.

Nguyen and Pham [97] examined the operation of the reflux ion concentration polarization (ICP) desalination system. The ion transport mode, the control factors of ion transport, and the ion transport in the desalination system were investigated in detail by numerical simulation. An accurate numerical solution model of ion transport was established. The process reduces the cost of the experiment. The actual reflux ICP desalination technology is optimized and shows good promise in seawater desalination.

### 4.2. Removal of Organic Pollutants by EME

Olasupo et al. [16] prepared a classical polymer inclusion membrane (PIM) for the removal of sulfamethoxazole antibiotics from aquatic samples by EME. The PIM was composed of the base polymer cellulose triacetate, the carrier Aliquat^®^336, and the plasticizer dioctyl phthalate. Sulfamethoxazole was removed with a transport efficiency of 100% at an extraction time of 15 h and a voltage of 50 V. Sulfamethoxazole was preconcentrated in river water and wastewater samples with preconcentrate coefficients of 22.9–24.1.

Restan et al. [98] reported the EME of removing sodium dodecyl sulfate (SDS) from samples that have been concentrated. The SLM was composed of 1.0% (*w*/*w*)Aliquat^®^336 in 1-nonanol and the driving force was 5 V. SDS was removed from a 100 µL aqueous sample using a 3 µL SLM, and the waste solution was 200 µL 0.01 mol/L NaOH in water. In 30 min of operation, EME eliminated 100% of samples with 0.1% SDS. It was found that SDS accumulated within the SLM during mass transfer and that replenishing the SLM or increasing the surface area helped get around some of these problems.

Roman-Hidalgo et al. [18] successfully extracted seven p-hydroxybenzoates and three fluoroquinolones simultaneously and selectively in a green electro-membrane extraction process using chitosan biopolymer membranes as the carrier. In this process, 10 mL of donor phase and 50 µL of acceptor phase were used, and the pH of both phases was 10. A voltage of 80 V was applied for 15 min extraction time. The enrichment factors were estimated to be ≥90 for parabens and ≥50 for three fluoroquinolones. The limits of detection were in the range of 0.2–1.1 µg/L. The method was successfully applied to remove pharmaceutical contaminants from water samples (rivers, lakes, and lagoons). The chitosan membrane used in the extraction process is a biodegradable and multifunctional carrier that can be a good substitute for the commonly used polypropylene material.

### 4.3. Resource Recovery by EME

Yang et al. [99] proposed an electromembrane reactor for recycling and resource recovery of flue gas desulfurization (FGD) residuals. First, by presenting the dimensionless groups, a mathematical model was created to characterize the mass balance of the proposed electromembrane reactor. Analysis of the model using dimensionless groups demonstrated that current density, flow pattern, and initial acid and base concentration were crucial factors in the optimization process, correlating with the outcomes of the experiments. This technology, integrated with existing FGD facilities, will enable the transfer of costly chemical processes to renewable resources and clean energy conversion processes.

Lin et al. [100] reported a Semi-dynamic EME for selective recovery of templates in molecular imprinting. The molecularly imprinted samples prepared by precipitation polymerization and native polymerization were used as the base template (propranolol) with recoveries of 61% and 55%, respectively. By using UV-Vis, HPLC-UV, and MS measurements, the selectivity of EME for template recovery from genuine samples was verified. This confirmed the great potential of EME for the one-step selective extraction and purification of ionizable compounds from complex samples.

Meng et al. [101] synthesized a novel PIM made of a compound carrier for bis(2-ethylhexyl) phosphate (D2EHPA) and TBP, and the PIM enabled the first realization of the EME of Li^+^. It has been proven that as the applied electric field voltage rises, the mass transfer rate of the system to Li^+^ increases dramatically. The driving force was 40 V, and the thickness of the membrane was 200 µm. The mass transfer mechanism of TBP and D2EHPA to Li^+^ was proposed.

Martoyan et al. [102] investigated a novel electro-membrane approach for ^7^Li isotope enrichment, which is a modified version of the amalgamation method for lithium isotope enrichment. The cations of the ^7^Li and ^6^Li isotopes moved in solution at various rates in response to an applied electric voltage between electrodes. The authors concluded that most of the ^6^Li isotopes were enriched in the mercury cathode, while the ^7^Li isotopes were concentrated on the top surface and in the working chamber. The mass spectrometer inductively coupled plasma-mass spectrometry performed analyses to determine the isotope ratio. The ^7^Li isotope separation coefficient was 1.178, confirming the possibility of enriching lithium isotopes using this method.

Trejo González et al. [103] selected a tubular renewable carbon-based material from sugar cane harvest residues as an electroactive membrane contactor to recover fresh water from brine. The finished carbon-based tubes had an outside dimension of 5.5 mm and an internal diameter of 2.5; the lengths employed in this study ranged from 10 to 50 mm. The driving force was 7 V, and the solute removal rate was higher than 99.4%. Membrane contactors have lower thermal conductivity than most commercial membranes and show promising applications in process thermal efficiency.

## 5. Conclusions

EME is an emerging green technology that is being developed for environmental analysis and remediation. EME reduces organic solvent and sample volume, shortens analysis time, and reduces operating costs. This review summarizes recent advances in the selection of membrane materials, modification of membranes, types of organic solvents, and applications in environmental pollutant detection and resource recovery. To offer a full knowledge of the utilization of EME in environmental applications, the impact of key process parameters has been highlighted. Operating factors including pH of acceptor/donor phases, stirring rate, applied voltage, temperature, and SLM type are supposed to enhance product recovery and selectivity. Under the appropriate conditions, EME technology provides sensitive determination of environmental pollutants, effective removal of pollutants from environmental wastewater, and rapid recovery of resources from the environment.

However, there are still a few issues that need to be addressed. Numerous EME setups have been created and taken into use for a variety of purposes over the last ten years. Most of the equipment is homemade and not commercially available. With the development of commercial and automated equipment, EME is sure to gain wider application in the future. Another appealing approach in this field is the automation and minimization of EME, even in complex matrices, based on novel and effective SLMs. In addition, more environmentally friendly and sustainable EME membranes are gaining increasing popularity among researchers. The majority of currently published studies only use agarose-based materials, primarily gel films, hence it’s critical to create novel, alternatives biodegradable materials. EME is mostly used in environmental analysis to identify contaminants in soil and water, while it is also starting to be applied to resource recovery and contaminant removal. Nowadays, with the increasing diversity of contaminants in original samples, the detection and removal of emerging contaminants (drugs, additives, dyes) also need to be given special attention.

In summary, this review acknowledged the potential and capability of EME to be used as a green extraction technique for the determination of organic pollutants and the sustainable removal of toxic pollutants. According to this review, additional in-depth studies that consider environmental factors more rationally than previous research works are still required.

## Figures and Tables

**Table 1:** Applications of EME for determination of inorganic ions.

Matrix	Analyte	SLM Composition	Detection System	Voltage (V)	Enrichment Factor and/or Recovery		LOD	Ref.
					EF	Recovery		
Wastewater samples, a nano silver suspension sample	Heavy metal cations	1-octanol containing 0.5% DEHP and 0.5% TEHP	AAS	60	-	>71.2%	-	[35]
Drinking water and environmental samples	Perchlorate	1-heptanol	CE-C^4^D	25	-	95.9–106.7%	0.25–0.35 µg/L	[36]
Water	Uranium (VI)	NPOE containing 1% DEHPA	Fluorescence measurements	80	>64.7	>54%	0.1 ng/mL	[37]
Drinking water samples	Perchlorate	1-pentanol and 1-hexanol	CE-C^4^D	150	30	-	-	[38]
Water samples	Cr(VI)	NPOE containing 15% DEHP	Digital image-based colorimetry	70	-	-	10 µg/L	[39]
Water samples	Cr(VI)	1-octanol	ETAAS	15	106	-	0.06 ng/mL	[40]
Water samples	Hg(II)	1-octanol	SWASV	60	-	89–97%	0.01 μg/L	[41]
Food supplement and drinking water samples	Iodide and Cr(VI)	Gel	Portable spectrometer	50	-	-	Iodide: 18 µg/L; Cr(VI): 5 µg/L	[42]
Wastewater sample	Basic drugs	3% of agarose gel	HPLC/UV	25	-	-	1.5–1.8 ng/mL	[43]
Water samples	Cr(VI) and Cr(III)	1-octanol	HPLC	30	-	31.1–47.2%	5.4 µg/L	[44]
Water samples	Cr(III) and Cr(VI)	Agarose gel	µPADs	65		Cr(VI): 58.8%; Cr(III): 83.3%	Cr(VI): 2.0 ng/Ml; Cr(III): 3.0 ng/mL	[17]
Water samples	As(III)	1-octanol with 2.5% DEHP	ASV	70	-	-	0.18 mg/L	[45]
Water samples	Cr(VI)	2-NPOE	UV-Vis	10–50	-	54.73%	-	[46]
Tap water and river water sample	Hg(II)	1-octanol with 2.0% DEHP	GFAAS	60	102–108	41–43%	0.5 µg/L	[47]
Drinking water and mineral water samples	Cr(III) and Cr(VI)	Cr(VI): Octanol-1; Cr(III): DEHP containing octanol-1 (0.7%)	µPADs	70	-	-	Cr(VI): 0.7 µg/L; Cr(III): 1.0 µg/L	[48]
Tap water and powdered milk samples	Heavy metal cations	1-octanol containing 0.5% bis(2-ethylhexyl) phosphonic acid	CE-C^4^D	75	-	-	2.5 × 10^−8^–2 × 10^−7^ mol/L	[49]
Pure water samples and water miscible organic solvents	Inorganic anions	1-heptanol	Ic	15	67–17	-	0.6–7.5 ng/mL	[50]
Water samples	As(V)	1-octanol containing 2.5% DEHP	UV-Vis	70	-	95–102%	1.5 ng/mL	[51]
Tap water, river water, and mineral water samples	Cr(VI) and Cr(III)	2-ethyl hexanol	ET-AAS	300	110	66	0.02 ng/mL	[52]
Mineral water, drinking water, tap water, and surface water	Cr(VI)	NPOE	UV-Vis	100	80	95–125%	-	[53]
Sea water	Bromide	Nitrobenzene containing 3% (*w*/*w*) BU6	CE-C^4^D	25	-	-	-	[54]
Tap, river and seawater	Pb(II)	20% DEHPA in 1-octanol	SWV	5	-	80.0%	9 × 10^−11^ mol/L	[55]
Water samples	thorium	1-octanol containing 5% DEHP	UV-Vis	90	50	-	0.29 ng/mL	[56]
Natural waters	Copper ions	dPKBH dissolved in 1-nonanol	AAS	95	-	-	0.004 mg/L	[57]
Spring water	Copper ions	NOPE containing 15.0% DEHP and 5.0% TEHP	µPADs	30	-	-	20.0 µg/L	[58]
Water samples	Zn(II)	Gel	FAAS	50	-	-	5.0 µg/L	[59]
Water and food samples	Lead	1-octanol	GFAAS	30	111	86.7–116.0%	0.011 ng/mL	[60]
Seawater	Nickel	DEHPA	GFAAS	25	180	-	23.3 ng/L	[61]
Tap water, food, and soil samples	Copper ions	1-octanol	RGB	10	-	-	0.1 µg/mL	[62]
Wastewater sample	Lead	1-octanol	RGB	9	-	97–102.6%	-	[63]

Note: Atomic absorption spectroscopy (AAS); Capillary electrophoresis with capacitively coupled contactless conductivity detection [CE-C^4^D]; Electrothermal atomic absorption spectrometry (ETAAS); Square wave anodic stripping voltammetry (SWASV); polyaniline (PANI); Gold nanoparticle-modified glassy carbon electrode (AuNP/GCE); High performance liquid chromatography (HPLC); Microfluidic paper-based analytical devices (µPADs); Anodic stripping voltammetry (ASV); Di-(2-ethylhexyl) phosphate (DEHP); di-2-ethylhexyl phosphoric acid (DEHPA); Electrothermal atomic absorption spectrometry (ET-AAS); Di-2-pyridyl ketone benzoylhydrazone (dPKBH); Ultra high resolution mass spectrometry (UHRMS); Diode array detection (DAD); Graphite furnace atomic absorption spectrometry (GFAAS); Electrospray ionization (ESI); Fluorescence detection (FLD); Flame ionization detection (FID); Ion chromatography (IC); Bambus[6]uril (BU6); Square wave voltammetry (SWV); Tandem mass spectrometry (MS-MS); Ultra-performance liquid chromatography (UPLC); Limit of detection (LOD); Enrichment factors (EF); “-” It means that the cited article does not mention or does not contain this item.

**Table 2:** Applications of EME for determination of organic pollutants.

Matrix	Analyte	SLM Composition	Detection System	Voltage (V)	Enrichment Factor and/or Recovery		LOD	Ref.
					EF	Recovery		
River water samples	Cationic quaternary ammonium and anionic chlorophenoxy acetic acid herbicides	Anionic carrier: di-(2-ethylhexyl)phosphoric acid; cationic carrier: aliquat^®^336	CE-C^4^D	3000	152–185	99.1–100%	0.3–0.4 µg/L	[68]
River and tap water samples	Antidepressants	[C_6_MIM] [PF_6_]	HPLC-UV	10	110–150	88.2–111.4%	0.1–0.4 µg/L	[69]
Water samples	Dicamba and 2,4-DB	1-octanol	CE	30	-	-	6.03 ng/mL	[70]
River water samples	Phenoxy acid herbicides	1-octanol	CE	7	-	-	10–15 ng/mL	[71]
Wastewater samples	Haloacetic acids and aromatic acetic acids	Toluene	HPLC-UV	200	-	87–106%	0.072–40.3 ng/L	[72]
Wastewater, urine, breast milk and spiked plasma samples	Acidic drugs	1-octanol	CE-UV	5	-	90–94%	1–3 ng/mL	[73]
Surface environmental waters	Parabens	1-octanol	HPLC-DAD	30	30–49	-	0.98–1.43 µg/L	[74]
Tap and river water samples	Carbendazim and thiabendazole	1-ethyl-2-nitrobenzene	CE	300	carbendazim: 26; thiabendazole: 50	-	Carbendazim: 2.3 μg/L Thiabendazole: 1.1 μg/L	[75]
Water samples	Imipramine, desipramine, citalopram and sertraline	1-heptanol	GC-MS	60	-	-	<0.25 ng/mL	[76]
Water samples	Basic degradation products of nitrogen mustard and VX	20% (*w*/*w*) DEHP in NPOE	UHPLC	100	-	15.7–99.7%	2–50 ng/mL	[77]
Drinking water	Five priority haloacetic acids	1-octanol	CZE-C^4^D	50	430–671	-	0.17–0.61 ng/mL	[78]
Water, urine, and plasma samples	Cyproheptadine and ketotifen	Polypyrrole and manganese dioxide	GC-FID	120	-	26.8–46.9%	Cyproheptadine: <0.7 ng/mL; Ketotifen: <1.1 ng/mL	[79]
Wastewater, plasma, and urine samples	Naproxen	1-octanol	HPLC-UV	20	-	66%	0.3 µg/L	[80]
Wastewater and urine samples	Verapamil, haloperidol, and rivastigmine	2-ethyl hexanol	LC-UV	170	-	-	2.0–3.0 ng/mL	[81]
Wastewater, plasma, breast milk, and urine samples	Alfentanil, sufentanil and methadone	1-octanol	GC	80	-	70.5–95.2%	0.6–1.5 ng/mL	[82]
Pure water, human plasma, wastewater, and breast milk samples	Acidic and basic drugs	Acidic drugs: 1-octanol; basic drugs: 2-ethyle hexanol	LC-UV	175	-	38.1–68%	0.3–1.5 ng/mL;	[83]
Wastewater and plasma samples	Alarelin, leuprolide, buserelin, and triptorelin	95% of 1-octanol and 5% DEHP	HPLC-UV	20	82–118	49–71%	0.2 ng/mL	[84]
Water samples, biological fluids, poppy capsules, and narcotic drugs	Thebaine	NPOE	HPLC-UV	300	90–110	45–55%	15 µg/L	[7]
Wastewater, human plasma, and human urine samples	Amlodipine, verapamil and clomipramine	2-ethyl hexanol	HPLC-UV	300	-	36.3–88.7%	-	[85]
Seawater samples	Chlorophenols	1-octanol	HPLC-UV	10	23	74%	0.1 ng/mL	[86]
Environmental water samples	Chlorophenols	1-octanol	GC-MS	50	2198		<0.005 µg/L	[87]
Water samples	Erythrosine and crystal violet	NPOE +5%DEHP and 1-octanol	RGB	50	-	≥94%	-	[88]
Produced water	Naphthenic acids	Decanol	UHRMS	200	-	-	0.10–0.13 µg/mL	[89]
Wastewater samples	β-lactam antibiotics	1-octanol	MEKC-UV	300	7.6–11.5	-	18 ng/mL	[90]
Wastewater samples	Cocaine	NPOE	CZE	100	-	89%	2.12 ng/mL;	[91]
Aqueous and blood samples	Five basic drugs	NPOE	LC-MS	75	-	75–87%	0.4 ng/mL	[92]

Note: Atomic absorption spectroscopy (AAS); Capillary electrophoresis with capacitively coupled contactless conductivity detection (CE-C^4^D); CZE with capacitively coupled contactless conductivity detection (CZE-C^4^D); Micellar electrokinetic chromatography with ultraviolet detection (MEKC-UV); 1-hexyl-3-methylimidazolium hexafluorophosphate ([C6MIM] [PF6]); molecularly imprinted polymer-coated multiwalled carbon nanotubes (MIP-MWCNTs); Electrothermal atomic absorption spectrometry (ETAAS); Square wave anodic stripping voltammetry (SWASV); polyaniline (PANI); Gold nanoparticle-modified glassy carbon electrode (AuNP/GCE); High performance liquid chromatography (HPLC); Microfluidic paper-based analytical devices (µPADs); Anodic stripping voltammetry (ASV); Di-(2-ethylhexyl) phosphate (DEHP); Electrothermal atomic absorption spectrometry (ET-AAS); Diode array detection (DAD); Graphite furnace atomic absorption spectrometry (GFAAS); Electrospray ionization (ESI); Fluorescence detection (FLD);“-” It means that the cited article does not mention or does not contain this item.

## Data Availability

Not applicable.
